# Prevalence and associated factors of overweight/ obesity among children and adolescents in Ethiopia: a systematic review and meta-analysis

**DOI:** 10.1186/s40608-018-0198-0

**Published:** 2018-07-09

**Authors:** Alemu Gebrie, Animut Alebel, Abriham Zegeye, Bekele Tesfaye, Aster Ferede

**Affiliations:** 1 Department of Biomedical Science, School of Medicine, Debre Markos University, P.O. Box 269, Debre Markos, Ethiopia; 2 Department of Nursing, College of Health Sciences, Debre Markos University, Debre Markos, Ethiopia; 3 Department of Public Health, College of Health Sciences, Debre Markos University, Debre Markos, Ethiopia

**Keywords:** Overweight, Obesity, Associated factors, Children, Adolescents, Ethiopia

## Abstract

**Background:**

Overweight and obesity can be defined as excessive and abnormal fat depositions in our body. They have become one of the emerging and serious public health concerns of the twenty-first century in low income countries like Ethiopia. Hence, the aim of this study was to determine the pooled prevalence and review associated risk factors of overweight/obesity among children and adolescents in Ethiopia.

**Method:**

The articles were identified through explicit and reproducible electronic search of reputable databases (PubMed, Google scholar, Science Direct, EMBASE, Cochrane library), and the hand search of reference lists of previous prevalence studies to retrieve more related articles. The 18 studies were selected based on a comprehensive list of inclusion and exclusion criteria. Data were extracted using a standardized and pre-tested data extraction checklist, and the analysis was done using STATA 14 statistical software. To assess heterogeneity, the Cochrane Q test statistic and *I*^*2*^ tests were used. Since the included studies exhibited considerable heterogeneity, a random effect model was used to estimate the pooled prevalence of overweight/obesity. Moreover, the risk factors of overweight/obesity were reviewed.

**Results:**

The combined pooled prevalence of overweight and obesity among children and adolescents in Ethiopia was 11.30% (95% CI: 8.71, 13.88%). Also, the separate pooled prevalence of overweight and obesity were 8.92 and 2.39%, respectively. Subgroup analysis revealed that the highest overweight/obesity prevalence among children and adolescents was observed in Addis Ababa, 11.94 (95% CI: 9.39, 14.50). Female gender of the children: 3.23 (95% CI 2.03,5.13), high family socioeconomic status: 3.16 (95% CI 1.87,5.34), learning in private school: 3.22 (95% CI 2.36,4.40), physical inactivity: 3.36 (95% CI 1.68,6.72), sweet nutriments preference: 2.78 (95% CI 1.97,3.93) and less use of fruits/vegetables: 1.39 (95% CI 1.10,1.75) have shown a positive association with the development of overweight/obesity among children and adolescents.

**Conclusion:**

The pooled prevalence of overweight/obesity among children and adolescents in Ethiopia is substantially high, and has become an emerging nutrition linked problem. Female gender, high family socioeconomic status, learning in private school, physical inactivity, sweet nutriments preference and less use of fruits/vegetables were found to be significantly associated with overweight/obesity.

## Background

Overweight and obesity can be defined as excessive and abnormal fat depositions in our body. They are major risk factors for several diet-linked non-communicable diseases like dyslipidemia, cardiovascular diseases (CVD), and type II diabetes mellitus [1–4]. Worldwide, overweight/ obesity is becoming one of the most challenging current health concerns with the worrisome rise in children and adolescents. Contemporary evidence revealed that the worldwide prevalence of overweight / obesity among children and adolescents were 13.5% [5]. In Africa, under-nutrition is the major nutritional problem affecting both children and adolescents. However, overweight/obesity is noticeably high with a prevalence of 8.5% in 2010 and predicted to be 12.7% by 2020. This situation pinpoints a double burden of malnutrition, and epidemiological as well as nutrition transition by virtue of several socioeconomic and demographic changes [5–7].

Studies showed that many factors can potentially be associated with overweight/obesity in children and adolescents [8, 9]. Those factors that are of maternal origin were socioeconomic status, education level, marital status, and smoking status during pregnancy. Gender of the children and adolescents, weight at birth, their birth rank, and residence were also the factors associated with overweight /obesity among them [10, 11]. Children and adolescents in developing countries are prone to sugar, high fat salts, energy rich foods and micronutrient-poor foods that are less costly and lower in nutrient quality. These dietary habits in combination with other factors result in substantial upsurge of overweight/obesity [12].

School based health education and promotion tactics such as enhancing physical activity among the children and adolescents, vegetables and fruits intake have been helpful to minimize overweight/obesity [13]. To solve this emerging health problem, Ethiopia incorporated the concern of overweight/obesity into the national nutrition program and launched an initiative to promote physical activity in the population [6]. Nevertheless, the efforts do not target children and adolescents in particular.

In different regions of Ethiopia, several independent and fragmented studies [14–30] were conducted in children and adolescents to assess the prevalence and associated factors of overweight / obesity, but there was a great variation and inconsistency of the findings among the studies. Hence, the aim of this review was to determine the pooled prevalence and associated factors of overweight/obesity in children and adolescents in Ethiopia. The results of the present study will elevate the need for policy makers, program planners, guardians or parents, clinicians as well as concerned stakeholders to give more emphasis for childhood overweight / obesity in the country. The review question is: What is the best available evidence on the prevalence and associated factors of overweight and/or obesity among children and adolescents in Ethiopia?

## Methods

### Literature search approach and study design

For its rigor, this study was guided by Preferred Reporting Items for Systematic Reviews and Meta-Analyses (PRISMA) [31]. The articles for this study were identified through comprehensive and reproducible electronic search of reputable databases (PubMed, Google scholar, Science Direct, EMBASE, Cochrane library), and the hand search of reference lists of previous prevalence studies to retrieve more related articles. The researchers also used the “related articles” option of PubMed and checked the reference lists of the original and review articles to detect more relevant publications. The search was independently performed by the two authors (AG, AA) using the following key terms: (a) population (preschool, children, schoolchildren, school aged, childhood, schooler, preadolescent, adolescent); (b) outcome (body composition, overweight, over nutrition, obesity, body constitution, weight status, body mass index, anthropometry; (c) study design (cross-sectional, prevalence, epidemiology, observational, pattern); and (d) location (Ethiopia and regions of Ethiopia) both in separation and in combination using the Boolean operator like “OR”, “AND” or “NOT”. Before searching the databases, the appropriateness of searching words was verified for retrieving the relevant articles. The literature search was limited to English language, and human study category. The literature records were managed using the EndNote X7 reference manager. The articles were searched from September, 2017 to November, 2017 and all the articles accessed until November, 2017 were included in this systematic review and meta-analysis.

### Selection of studies

#### Inclusion criteria

The two investigators independently and reproducibly assessed the contents of each of the identified studies (AG and AA). Those articles which met the following criteria were included in the study.

**Population:** Articles conducted among children and adolescents (age < 20 years) were considered.

**Study area:** Only articles conducted in Ethiopia were considered.

**Study design**: Original studies that reported the prevalence and associated risk factors of overweight and/ or obesity, measured objectively by trained personnel, among children in Ethiopia were considered.

**Language**: Only articles published in English language were considered.

**Publication condition**: Studies that fulfilled the eligibility criteria were included regardless of their publication status (published, unpublished and grey literature, etc.)

#### Exclusion criteria

After screening the abstracts and the full texts of the articles, the three researchers (AG, AA and AF) carried out the data extraction independently and blindly. Articles with methodological problems were excluded by the three independent researchers after reading the full text as well as abstracts. The articles the full texts of which we were not able to fully access or failed to contact their primary authors were excluded from this review because of incomplete data.

#### Data abstraction and critical appraisal of the studies

The two researchers independently extracted all the necessary data using a standardized and pre-tested data extraction checklist. The necessary data extracted from the articles included: first author of the study, region in Ethiopia where the study was carried out, the particular area where the study was conducted, study design, publication year of the study, sample size, response rate, and prevalence of overweight/obesity. Any sort of discrepancies between the researchers on the data extracted were solved through discussion and consensus as well as through involvement of the third reviewer (AZ).

The reviewers employed the Newcastle-Ottawa quality assessment tool Scale adapted for cross-sectional studies so as to appraise the qualities of the studies [32]. The tool is composed of three important indicators. The first part is graded from five points (stars) and evaluates the methodological qualities of the studies. The second part has three stars and assesses the comparability of the studies. The last section of the tool is graded from two points and measures the quality of the original studies in terms of their statistical analyses. By using the tool as a guiding protocol, the two authors (AG and AA) appraised the qualities of the primary studies independently.

The qualities of the studies were assessed by using the following indicators; those with medium (fulfilling 50% of quality assessment criteria) or high quality (≥6 out of 10 scales) were considered for inclusion in the meta-analysis. Taking the mean score of the two reviewers, differences of their assessment results were determined.

#### Operationalization of the outcomes of the review

Firstly, the percentage of overweight/obesity among children and adolescents was the foremost outcome of the meta-analysis. The second outcome of the study was to examine the factors that are associated with overweight/obesity among the study subjects. The prevalence was obtained by dividing the number of children and/or adolescents who are either overweight or obese to the total number of children and/or adolescents who have been included in the study (sample size) then multiplied by 100. The association between overweight/obesity and the factors were quantified by odds ratio. The odds ratio was calculated from the two by two table reports of the primary studies.

#### Data analysis/synthesis of results

After the relevant data had been extracted from the studies by using Microsoft excel 2016 format, the authors then analyzed the results by using STATA version 14.0 (STATA Corporation, College Station Texas) software. The original studies were summarized and presented by using a table and the forest plot. The authors computed the standard error of prevalence of overweight/obesity for each original study by using binomial distribution formula. We explored the potential heterogeneity among the reported prevalence of the studies using I^2^ test and Cochrane Q statistics [33]. Since the test statistics revealed that there was a considerable heterogeneity [34] among the studies (I^2^ = 96.9%, *p* = 0.000), a random effects model was used to estimate the Der Simonian and Laird’s pooled effect. We also undertook univariate meta-regression analysis taking publication year of the studies and the sample size to detect the potential source(s) of variation but both of them were found to be statistically insignificant, (*p* = 0.65 and *p* = 0.45 respectively). Possible publication bias was also objectively examined using Egger’s weighted correlation and Begg’s regression intercept tests at 5% significant level respectively [35, 36]. The test results showed there is a significant publication bias (*p* = 0.000), and therefore the final effect size was determined by applying Duval and Tweedie’s Trim and Fill analysis in the Random-effects model. In addition, to minimalize the random variations between the point estimates of the original studies, subgroup analysis was carried out based on region of studies and publication year.

## Results

### Identification of eligible studies

**Fig. 1:**
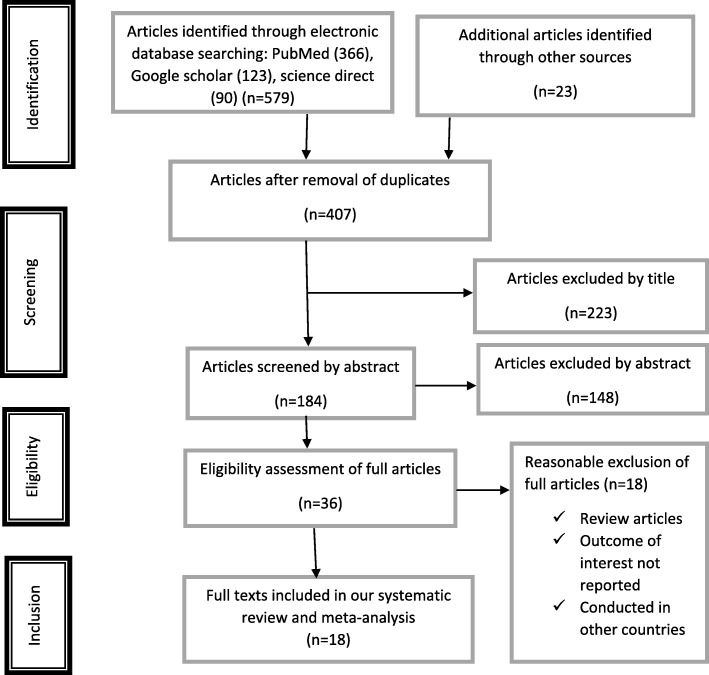
Flow chart diagram describing selection of studies for the systematic review and meta-analysis of prevalence and associated factors of overweight/obesity among children and adolescents in Ethiopia, 2018 (identified, screened, eligible and included studies). Articles may have been excluded for more than one reason

From the outset, we searched a total of 602 records by the electronic search through a search engine of MEDLINE/PubMed, Google scholar, science direct, EMBASE, Cochrane Library and reference lists of previous related studies to retrieve more related articles. Since there were duplications in the records, 195 of them were removed from the inclusion. After assessing the abstracts and titles, the remaining 407 retrievals, 371 records were excluded since they were not relevant for this meta-analysis in terms of outcome the study is interested. Then, 36 full text studies were considered and assessed for eligibility based on the pre-set eligibility criteria. Finally, 18 studies were considered to be eligible and included in this systematic review and meta-analysis (Fig. 1).

From a total of 36 full text studies accessed, we removed twelve of them because they were review articles, and /or conducted in other nations which are not the location of interest of the study like: USA [37, 38], Russia [39], Japan [40], Yemen [41], Nigeria [42–45], South Africa [46], Egypt [47] and Morocco [48]. Moreover, six full text studies [49–54] that have been carried out from different parts of Ethiopia were excluded because their outcome measures were not prevalence of overweight/obesity in children and they were conducted in the adult population which is not the population of interest of the this study.

### Description of original studies

Table 1 summarizes the descriptive characteristics of the 18 studies included in this systematic review and meta-analysis. All the studies were cross sectional by design, and conducted in different parts of Ethiopia with a sample size ranging from 174 in Adama, Oromia region [26] to 9880 in a national survey [55]. The included studies have been conducted from 2010 to 2017. In the current systematic review and meta-analysis, a total of 19,031 children and adolescents were included to estimate the pooled prevalence of overweight/obesity.

**Table 1:** Characteristics of 18 studies reporting the prevalence of overweight/obesity among children and adolescents in Ethiopia included in the current systematic review and meta-analysis, 2018

Region	Area	Author	Publication year	Sample size	Response rate (%)	Quality score (10 pts)	Prevalence (95% CI)
Addis Ababa	Addis Ababa	Gebremichael et al. [22]	2017	463	96.9	6	12.74 (9.71,15.78)
	Bole subcity	Askal et al. [19]	2015	828	97.9	7	9.78 (7.76,11.81)
	Addis Ababa	Dessalegn and Robel [29]	2016	390	96.3	6	18.21 (14.38,22.03)
	Addis Ababa	Mulugeta et al. [30]	2016	446	97.8	6	15.25 (11.91,18.58)
	Arada subcity	Alemu et al. [23]	2014	800	100	7	9.38 (7.36,11.39)
	Addis Ababa	Yoseph et al. [16]	2014	1024	100	6	8.50 (6.79,10.20)
Amhara	Bahirdar	Tadesse et al. [21]	2017	462	97	8	6.93 (4.61,9.24)
	Gondar	Sorrie et al. [18]	2017	500	99.2	8	13.80 (10.78,16.82)
	Gondar	Gebremedhin et al. [17]	2013	791	98.9	7	5.94 (4.29,7.59)
	Bahirdar	Zelalem et al. [24]	2015	431	95.6	8	16.71 (13.18,20.23)
Dire dawa	Dire dawa	Desalew et al. [14]	2017	448	98.2	7	20.54 (16.80,24.28)
Ethiopia	Ethiopia	EDHS [55]	2011	9880	NR	7	3.00 (2.66,3.33)
Harari	Babile	Kedir Teji et al. [28]	2016	547	91	6	5.85 (3.88,7.82)
Oromia	Ambo	Mesert Yetubie et al. [25]	2010	425	NR	7	8.71 (6.03,11.39)
	Jimma	Dessalegn et al. [15]	2017	510	93.4	8	13.33 (10.38,16.28)
	Adama	Wakayo et al. [26]	2016	174	98	7	10.92 (6.29,15.55)
SNNP	Hawasa	Woldie and Belachew [20]	2014	358	100	7	10.61 (7.42,13.81)
	Hawasa	Teshome et al. [27]	2013	554	97	5	15.70 (12.67,18.73)

The 18 studies have been conducted in the six regions of Ethiopia: about one third (six) of the included studies were carried out in Addis Ababa: Addis Ababa [19, 21–23, 29, 30], Dire dawa [14], Amhara [17, 18, 21, 24], Harari [28], Oromia [15, 25, 26], Southern Nations, Nationalities and peoples’ region (SNNPR) [20, 27] and one nationwide survey study [55]. Whereas the highest prevalence of overweight/obesity (20.54%) was reported in Dire dawa [14], the nationwide survey study [55] reported the lowest prevalence (3%) of the problem. Furthermore, the original studies included in this meta-analysis and reporting response rate had a response rate that ranges from 91 to 100% showing that all the studies had good response rate.

Concerning the quality of the articles: only one [29] of the 18 studies was unpublished article and the studies included in the meta-analysis were identified by exhaustive search from reputable journals like PubMed. Blinded reviewers re-evaluated all the studies before analysis and the articles were found fit for their quality (quality score ranged from 5 to 8 out of 10 points).

### Meta-analysis and meta-regression

**Fig. 2:**
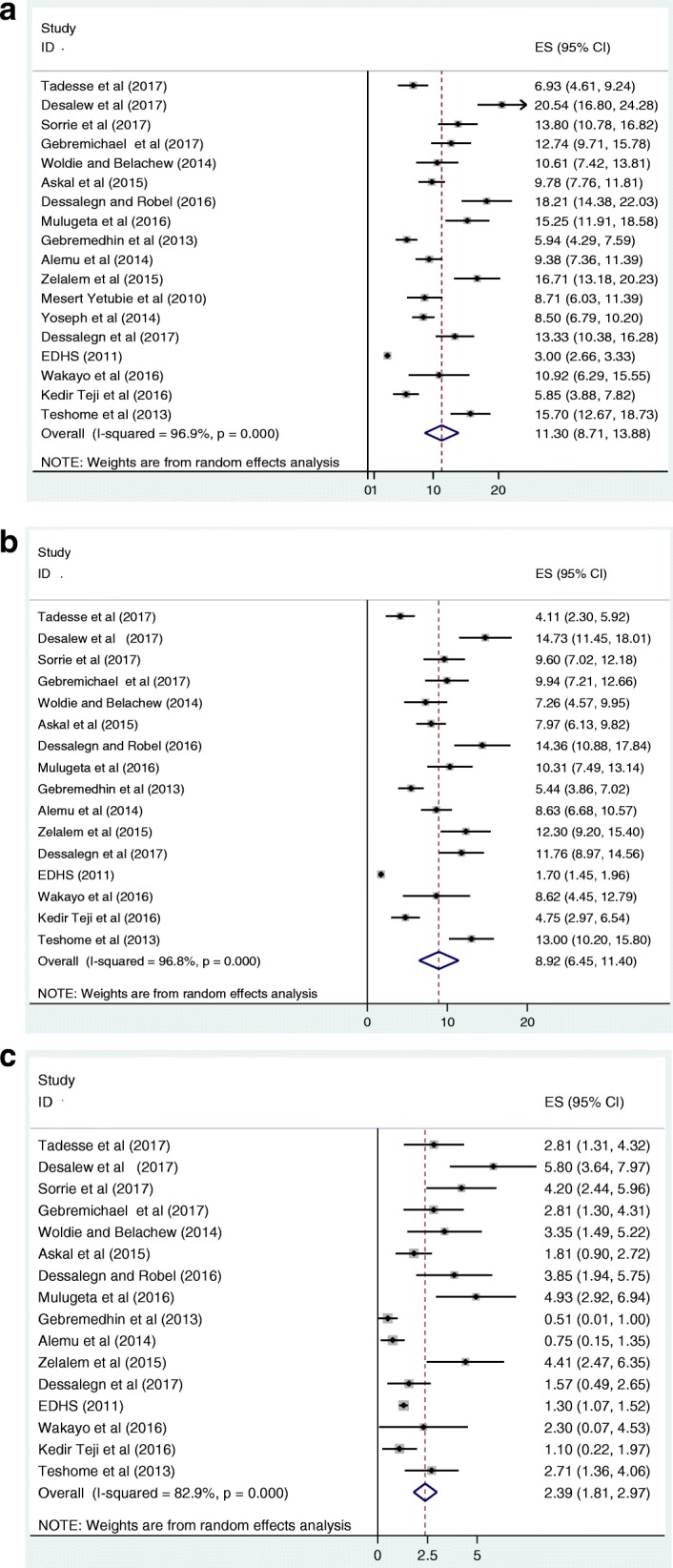
Forest plot of the pooled prevalence of overweight and obesity in children and adolescents, 2018 (**a**: The combined pooled prevalence of overweight/obesity; **b**: The pooled prevalence of overweight; **c**: The pooled prevalence of obesity)

As it is depicted in the forest plot of 18 included studies below (Fig. 2a), the combined pooled prevalence of overweight and obesity among children and adolescents in Ethiopia was 11.30% (95% CI: 8.71, 13.88%). Also, from the 16 studies, the separate pooled prevalence of overweight and obesity were 8.92 and 2.39%, respectively (Fig. 2b and c). Considerable heterogeneity [34] was observed among the 18 studies and detected by I^2^ statistic (I^2^ = 96.9, *p* value < 0.000). As a result, the DerSimonian and Laird random-effects method, which gives more conservative estimate, was used to estimate the overall pooled prevalence of overweight and obesity among children and adolescents. To determine the likely sources for the variation, we checked the potential factors associated with the prevalence variation, publication year and sample size by using univariate meta-regression models but both of them were found to be statistically insignificant for the variation (Table 2). Egger’s and Begg’s tests showed that there is a statistically significant publication bias, (*p* = 0.000) and (*p* = 0.002) respectively. Hence, we performed Trim and Fill analysis so as to adjust the final pooled prevalence of overweight/obesity among the subjects.

**Table 2:** Related factors with the heterogeneity of overweight/obesity prevalence among children and adolescents in Ethiopia in the meta-analysis (univariate meta-regression)

Variables	Coefficient	*P*-value
Year of publication studies	0.2866022	0.817
Sample sizes of the studies	−0.0005473	0.411

### Subgroup analysis

We have also performed subgroup analysis based on the region where the studies were carried out and year of publication of the studies to assess possible causes of considerable heterogeneity. As per the result, the highest prevalence of overweight/obesity among children and adolescents was observed in Addis Ababa, where most of the studies have been conducted, with a prevalence of 11.94 (95% CI: 9.39, 14.50) followed by regions classified (in this study) as others, 10.97% (95% CI: 5.09, 16.85) and Oromia region 10.94% (95% CI: 7.86, 14.02) (Table 3). Regarding year of publication, the prevalence of childhood overweight/obesity was significantly higher in studies which have been published since 2014, 12.95% (95% CI: 10.17, 15.73) compared to those articles published before 2014, 8.70% (95% CI: 5.40, 11.99) (Table 3).

**Table 3:** Results from subgroup analysis of the prevalence of overweight/obesity among children and adolescents in Ethiopia, 2018 (*n* = 18)

Variables	Characteristics	Number of studies	Prevalence with 95%
Region	Addis Ababa	6	11.94 (9.39, 14.50)
	Amhara	4	10.66 (5.92, 15.41)
	Oromia	3	10.94 (7.86, 14.02)
	Others	5	10.97 (5.09, 16.85)
Year of publication	≤ 2014	7	8.70 (5.40, 11.99)
	> 2014	11	12.95 (10.17, 15.73)

### Associated factors of overweight/obesity among children and adolescents

**Fig. 3:**
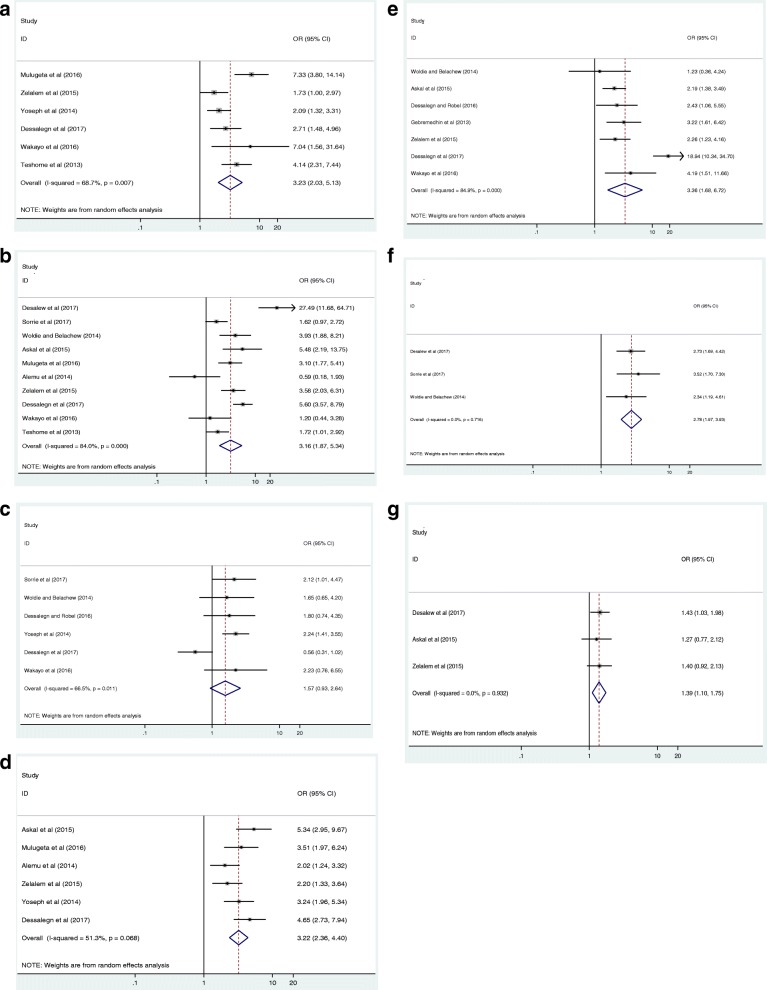
Forest plot showing the pooled odds ratio of the associations between overweight/obesity and its purported associated risk factors among children and adolescents in Ethiopia (**a**: Sex, **b**: Family income, **c**: Family education, **d**: School type of children, **e**: Physical activity, **f**: Use of sweet food, **g**: use of fruits and vegetables)

We have comprehensively reviewed and meta-analyzed the associated risk factors of overweight/obesity among children and adolescents by using thirteen relevant studies [14–20, 23, 24, 26, 27, 29, 30] from the eligible articles included in this study. Sex of the children, family income, family educational status, type of school the children attend, physical activity status, habit of using sweet nutriments, use of fruits and vegetables were found to be worth reviewing and meta-analyzable. They were associated with overweight/obesity among children and adolescents in Ethiopia (Fig. 3). We have also performed sensitivity analysis for each of the factors but none of the studies revealed significant difference.

The pooled effect size of six studies showed that female children and adolescents were 3.23 times more likely to be overweight/obese than their male counterparts, odds ratio 3.23 (95% CI 2.03,5.13) (Fig. 3a). The pooled result of ten studies also revealed that children and adolescents from high income families were 3.16 times more likely to be overweight/obese as compared to those children with middle and low-income families, odds ratio 3.16 (95% CI 1.87,5.34) (Fig. 3b). Those children and adolescents whose families are not illiterate (educated) were 1.57 times more likely to have overweight or obesity compared to those whose families are illiterate, odds ratio 1.57 (95% CI 0.93,2.64) (Fig. 3c**)**.

We have also found that children and adolescents attending at private schools were 3.22 times more likely to develop overweight/obesity as compared to those attending at governmental schools, odds ratio 3.22 (95% CI 2.36,4.40) (Fig. 3d). Physically inactive children and adolescents were also 3.36 times more to be overweight/obese than those children who were physically active, odds ratio 3.36 (95% CI 1.68,6.72) (Fig. 3e). In addition, children and adolescents who had the habit of using sweet food stuffs were 2.78 times more likely to suffer from the emerging nutritional problem of overweight and obesity as compared to those children who have seldom used sweet food items, odds ratio 2.78 (95% CI 1.97,3.93) (Fig. 3f). Moreover, the results of three studies showed that infrequent consumption of fruits and vegetables was a risk factor for the development of overweight/obesity among children, odds ratio 1.39 (95% CI 1.10,1.75) (Fig. 3g).

## Discussion

Overweight/obesity, an emerging nutritional problem in developing countries, increases the burden of nutrition related diseases and have far-reaching consequences on economic growth of countries [4–6, 12]. Evidences have revealed that nutrition-linked non-communicable diseases are significantly in upsurge over time. In contrary to previous concerns that mainly focused on the issue of under nutrition, overweight/obesity is now emerging as a nutrition related public health burden. The problem is debilitating and a double burden in low income communities in different parts of Africa most importantly in Ethiopia. Therefore, the result of the present study is of paramount importance in pinpointing the emerging problems of nutrition-linked concerns particularly among children and adolescents in the country.

A systematic review and meta-analysis has not yet been carried out to estimate the pooled prevalence of overweight/obesity, and review its associated factors among children and adolescents in Ethiopia. However, about 18 cross sectional studies, which are relevant for this research question, have been conducted in different parts of Ethiopia. Hence, the aim of this study was to estimate the pooled prevalence of overweight/obesity as well as to identify and review the risk factors that are associated with it among the study subjects. Using those relevant studies, the result of this meta-analysis showed that the combined overall prevalence of overweight and obesity, as per WHO 2007 definition, was 11.30% (95% CI: 8.71, 13.88%) among children and adolescents in the country. This prevalence is substantially high even comparable with the results reported for some developed countries (8.7%) a couple of years ago, and higher than the global prevalence (7%) [56, 57]. Despite methodological differences, nutritional patterns and the availability of recreational facilities may be attributed for the variation in the results, the findings of this study clearly indicate that there is a nutrition transition, and overweight/obesity is becoming a growing problem and a double burden in the country.

Also, the subgroup analysis of this meta-analysis revealed that the prevalence of childhood overweight/obesity varies across the regions of Ethiopia. The prevalence was highest in children and adolescents of Addis Ababa as compared to other regions of the country. This could be due to better access to high calorie diets because of better socioeconomic status of the population in the capital compared to other regions of the country [4]. The prevalence of overweight/obesity was also significantly higher in those studies published since 2014 as compared to studies published before 2014. This shows that childhood overweight/obesity is increasing alarmingly in the country [57].

We reviewed and meta-analyzed the risk factors related with overweight/obesity among children and adolescents that have been addressed by the relevant studies. The risk of developing childhood overweight/obesity among females was higher than males in this review. Though they are not reviews, the result was congruent with studies conducted in African countries that female subjects were more likely to be at risk of having overweight/obesity compared to the male ones [58, 59]. But the result is in contrary to studies conducted from other countries [60–62] in which overweight/obesity was more prevalent in males than females. However, the prevalence in most developing countries is more in females which is true for this meta-analysis too. This may be explained that there is a biological difference in energy need for males and females in relation to rate of growth and timing of sexual maturation [63]. Males are also more physically active than females particularly during childhood [64]. In addition, in developing countries such as Ethiopia, girls usually stay at home for long period and there is a cultural influence not to move much from place to place than boys leading to physical inactivity and ultimately the development of overweight and obesity.

Children and adolescents from families with high monthly income were more likely to be overweight/obese as compared to those from families with low and moderate income. This is in line with a study done in Saudi Arabia [65], but in contrary to studies conducted in Germany and Korea [66, 67] where lower income was found to significantly increase the risk of being overweight/obese in the study subjects. This could be due to the reason that adolescents from socioeconomic status families have access for fat rich foods and follow sedentary life style. On the other hand, patterns of high expenditure of energy among the poor families and cultural attitude towards a larger body size tendency could also contribute to the positive relations observed. In lower-income countries like Ethiopia childhood overweight/obesity has been considered as a sign of better social status, and healthiness [68].

In contrast to cross sectional studies conducted in developed countries like Brazil and Iran [69, 70], the educational level of families was positively associated with overweight/obesity in the subjects but not statistically significant. Those children and adolescents whose mothers had primary educational level and above were more likely to be overweight/obese as compared to mothers with no education. This shows that educated mothers in developing countries fail to make healthy choices regarding food stuffs like the incorporation of fruits and vegetables in the diet.

There was a remarkable difference in the prevalence of overweight/obesity between government and private schools where children attended. The odds of having overweight/obesity was higher among children attending at private schools than those at governmental schools. This is in trajectory with studies conducted in India [71], Yemen [72], Saudi Arabia [73], Kenya [74] and Burkina Faso [66]. Often, children attending private schools are from families of higher socioeconomic status. Therefore, they are exposed to unhealthy nutritional patterns such as highly-processed and fast foods, more animal products as well motorized lifestyle compared to those children attending government schools.

In addition, adolescents not engaged in physical activities were more likely to be overweight and obese compared to those who were doing physical activities. The finding is concordant to other cross-sectional studies [75, 76]. The possible explanation could be because doing physical exercise burns off body fat (negative energy balance) leads to less risk of overweight/obesity. Children who consumed sweetened foods were more likely to be overweight/obese compared to those who didn’t consumed. This was congruent with WHO report of different studies done in Europe [77], Egypt [78], Kenya [79]. This could be reasoned out that sweet food products are calorie rich and have a greater acceptance by children and adolescents resulting in a positive energy balance to them. Moreover, the results of three studies showed that infrequent consumption of fruits and vegetables was a risk factor for the development of overweight/obesity among children and adolescents.

### Strengths and limitations of the study

The strengths of this study were the use of multiple reputable databases to exhaustively and explicitly search eligible literatures as well as uniform and reproducible extraction of data using a preset and pretested checklist so as to minimize errors. This systematic review and meta-analysis also included studies from different regions of the country. However, the study may have some potential limitations for it is restricted to articles published in English language. In addition, by virtue of the cross-sectional nature of the studies reviewed, temporal relationship cannot be established between the factors and overweight/obesity. Despite the incorporation of studies from different parts of the country, the representativeness of the population is not so strong as it could have been. Therefore, one had better interpret the findings of this systematic review and meta-analysis in context of the inherent limitations of both the original studies and the present review.

## Conclusions

This meta-analysis revealed that the prevalence of overweight/obesity among children and adolescents in Ethiopia is substantially high, and has become an emerging nutrition linked problem. Unless successful preventive measures are taken, the problem may continue on upsurge in the future. Female gender of the children, high family socioeconomic status, learning in private school, physical inactivity, sweet nutriments preference and less use of fruits/vegetables were found to be significantly associated with overweight/obesity among the study subjects. We have recommended that integrated nutrition education program has to be successfully implemented in schools as well as in communities with existing health extension programs. Healthcare providers and policymakers should also give more emphasis on the design and implementation of preventive policies to control the rising prevalence of childhood overweight/obesity in Ethiopia.

## Abbreviations

PRISMA: Preferred Reporting Items of Systematic Reviews and Meta-Analysis
SNNPE: Southern Nations, Nationalities and peoples of Ethiopia
USA: United States of America
